# Food Insecurity Among Transgender and Gender Nonconforming Individuals in the Southeast United States: A Qualitative Study

**DOI:** 10.1089/trgh.2018.0024

**Published:** 2019-03-06

**Authors:** Jennifer Russomanno, Joanne G. Patterson, Jennifer M. Jabson

**Affiliations:** Department of Public Health, The University of Tennessee, Knoxville, Tennessee.

**Keywords:** discrimination, food insecurity, gender nonconforming, health outcomes, poverty, transgender

## Abstract

**Purpose:** Transgender and gender nonconforming (TGNC) people experience high rates of poverty, joblessness, and homelessness, which drive risk for food insecurity. TGNC people also face discrimination due to transphobia and cissexism, which may contribute to these drivers. Minimal empirical evidence describes experiences with food insecurity among TGNC people. This project investigated food insecurity among TGNC people and how these experiences relate to their physical and mental health.

**Methods:** Semistructured telephone interviews were conducted with 20 TGNC people residing in the Southeast United States (U.S.), recruited through social media. Interviews were transcribed and qualitatively coded.

**Results:** TGNC people reported living in extreme poverty. They described how the conservative sociopolitical climate of the Southeast United States made it difficult to find and maintain employment, which was a primary driver of food insecurity. Participants experienced discomfort seeking food assistance due to discrimination and concern for reducing emergency food availability for people in greater need. Stress from unemployment and underemployment, inadequate food supplies, and discrimination was reported as a contributor to poor physical and mental health, and weakened support systems.

**Conclusion:** Poverty and food insecurity erode TGNC people's physical and mental health and support systems. TGNC people faced substantial barriers—including unemployment and underemployment and multilevel discrimination—which prevented them from affording adequate food. Public health solutions include implementing employment nondiscrimination policy to protect TGNC people in the workplace and building relationships between local food pantries and LGBT organizations to create safer environments for all persons in need of food assistance.

## Introduction

Food security is defined by the United States Department of Agriculture (USDA) as “access by all people at all times to enough food for an active, healthy life.”^1^ An estimated 11.8% of American households are food insecure, meaning that their access to adequate food is limited by a lack of money and other resources.^1^ People who are food insecure often have diets rich in cheaply processed, energy-dense foods including refined grains, added sugars, and added saturated fats.^2^ These dietary deficiencies contribute to several diet-related chronic diseases, including hypertension, heart disease, and diabetes.^2,3^

Some subgroups of the population are more likely to be food insecure than others.^4^ For example, adults living in households with incomes at or below 185% of the federal poverty level are more likely to have high food insecurity than those above this income threshold.^1^ Food insecurity is a dynamic, managed process that has been linked to a number of economic, structural, and psychosocial factors, including unemployment and underemployment, poverty, high housing costs, access to food, household stress, receipt of local, state, or federal food assistance, and health care expenses.^5–7^

Transgender and gender nonconforming (TGNC) people face very high risks for poverty, joblessness, homelessness, and stress.^8,9^ TGNC people comprise individuals of varied identity labels, including, but not limited to transgender, gender nonconforming, nonbinary, gender fluid, genderqueer, or gender expansive. A TGNC-identified person's gender identity differs from their sex assigned at birth and/or exists beyond the gender binary (i.e., social construction of gender as strictly “man” or “woman”) and from the majority of the surrounding society. Conversely, a cisgender person's gender identity corresponds with their sex assigned at birth and exists within the gender binary.^10^

To date, the 2015 U.S. Transgender Survey (USTS)^9^ is the largest and most comprehensive survey documenting the experiences of transgender people in the United States (U.S.) and is among the first sources of evidence to describe poverty, joblessness, and homelessness among this group. Among USTS respondents, nearly one-third (29%) of transgender people were living in poverty, compared to 12% of the general population. Furthermore, 15% of respondents were unemployed, three times higher than the at-current U.S. unemployment rate (5%). Nearly one-third (30%) of USTS respondents experienced homelessness during their lifetime, and 12% experienced homelessness in the year before survey completion because of their transgender identity.^9^ Given these extreme economic hardships, food insecurity is an urgent issue for TGNC people; however, little is known about food insecurity in this group.

Generally, experiences of food insecurity are associated with negative psychosocial factors, including anxiety, stress, shame, and humiliation.^5,11,12^ As such, food insecurity is characterized as a physically and emotionally distressing experience that erodes mental health and long-term quality of life.^12^ While negative psychosocial suffering is well documented in the general population, we have little evidence exploring how food insecurity affects TGNC people, and more specifically, how gender identity intersects with issues of food insecurity.

In addition to stress and anxiety induced by food insecurity, the TGNC population faces an additional burden of minority stress. Minority stress is a unique form of stress that exists in addition to the daily hassles and life events experienced by all people. Minority stress is chronic and ongoing, cumulative, and institutional, as it is built into how organizations and social phenomena function and exists beyond the control of the individual or subgroup it targets.^13^

Minority stress has the capacity to influence TGNC people's lives at multiple socioecological levels.^13,14^ Intrapersonally, minority stress can have psychosocial repercussions, including social anxiety, depression, and self-acceptance.^15^ Interpersonally, TGNC people face rejection by peers, family, and romantic partners, and social isolation. They may also experience interpersonal prejudice, violence, and threat of violence.^13^ At a community level, TGNC people risk discrimination by organizations, including unemployment or underemployment, refusal of care by health care systems, and exclusion from community and religious groups.^9,13^ On a societal level, TGNC people face stigma and marginalization by discriminatory policies and laws that target this group.^16^ Multilevel minority stressors are likely to intersect with food insecurity, as they may contribute to or amplify the negative consequences of food insecurity in the TGNC population.

TGNC people living in the Southeast may be particularly at risk for increased stigma, marginalization, and discrimination within their communities due to the region's sociopolitically conservative and religious climate.^9,17^ Many Southeastern states have not passed nondiscrimination laws protecting TGNC people and some have enacted policies that restrict TGNC people's rights.^18^ Moreover, of the 12 states defining the Southeast, 9 report a prevalence of food insecurity higher than the national average.^19,20^ As such, TGNC people living in the Southeast are experiencing some of the worst contexts for food insecurity and gender-based discrimination.

The purpose of this study was to understand food insecurity experienced by TGNC people in the Southeast U.S. We sought to answer the following questions: What experiences do TGNC individuals living in the Southeast U.S. have with food insecurity? How does food insecurity relate to health outcomes for TGNC individuals living in the Southeast United States? Answering these questions will inform future policies and/or community-based interventions that break down barriers and promote high food security in TGNC groups.

## Methods

This project was approved by the University of Tennessee Institutional Review Board (IRB-16-03275-XP).

### Paradigmatic framework

Given the lack of published evidence of food insecurity in TGNC individuals, it was imperative to gain in-depth insight on how food insecurity is affecting this population from their perspectives. Therefore, this study was informed by the paradigmatic framework of constructivism.^21^ Interview questions were intentionally constructed in a semistructured, conversational tone that left room for participants to guide the interview from their own perspectives of experiences with food insecurity.^21^

### Methodology

We focused on the experiences of food insecurity among a particular group of people (TGNC living in the Southeast); consequently, we used an instrumental case study approach to focus more on the phenomena being investigated rather than on individual cases themselves.^22,23^

### Research team

The three-person research team consisted of cisgender females who all identify as members of the LGBTQ community. The Principal Investigator (PI) (J.R.) and co-author (J.P.) are both doctoral candidates, while J.J. is the faculty advisor. All three team members' research focuses on health and/or food insecurity of marginalized populations, including LGBTQ people. J.R. conducted a majority of participant interviews (75%), while J.P. conducted 10%, and J.J. conducted 15%. All interviewers underwent extensive training in interviewing and qualitative data collection, methods, and analysis before this project.

### Participants

Purposeful, criterion-specific sampling^22^ was used to recruit members of this hard-to-reach population.^24^ Eligibility criteria were as follows: speak and understand English, be older than 18 years, identify as TGNC, self-report issues of food insecurity within the past 12 months by completing the USDA-approved Food Security Module “Short Form” 6-item scale,^25^ and reside in 1 of the 12 Southeast states of the United States: Alabama, Arkansas, Florida, Georgia, Kentucky, Louisiana, Mississippi, North Carolina, South Carolina, Tennessee, Virginia, or West Virginia.

Informed consent was obtained from all participants before participation in a prescreening questionnaire that assessed level of food security as defined by the USDA food security module.^25^ Prescreening questionnaires were sent by e-mail by the PI. Participants with a raw score of 2 through 6 (low to very low food security, or, food insecure) were eligible to participate. Those who scored 0 or 1 (high to marginal food security) would be excluded from participation. All individuals who contacted us to participate scored a 4 or greater on the USDA module. None was excluded based on scores.

### Recruitment strategy

Participants were recruited online by LGBT-centric Facebook groups and Facebook advertisements. Advertisements targeted individuals who were 18–50 years of age, resided in the Southeast United States, and had interests in LGBT-related topics. Each advertisement included a brief introduction to the study and PI contact information. In total, two paid advertisement campaigns were run over 12 days. Twenty-two people responded to recruitment requests. Twenty participants were interviewed and two were lost to follow-up between eligibility screening and scheduled interview. Three participants contacted us after recruitment was closed to participation; they were not included nor interviewed.

Study participation was voluntary. Each participant was assigned a unique identification number to maintain confidentiality. Participants were compensated with a $25 electronic gift card for completing a qualitative interview.

### Procedures

We used the COREQ 32-item checklist^26^ to identify important study characteristics for reporting—including data collection, analysis, sampling methods, and theme development. Participants completed a one-time, semistructured interview in person or by telephone. A semistructured interview guide (Table 1) informed answers to the study's two research questions. Interview questions were developed based on research questions, a pilot-test interview, known consequences of food insecurity in the general population, and constructs from minority stress theory. Based on our inclusion criteria, we knew participants were experiencing food insecurity on some level; therefore, presupposition questions^27^ were developed to ensure potentially difficult or embarrassing life experiences were accurately recorded. To confirm interview guide quality and ensure questions were appropriate and sensitive to TGNC people, the PI pilot tested the interview guide with a self-identified transgender male who scored a 6 for food insecurity.

**Table 1.** Semistructured Interview Guide

*Warm up*
1. To begin, as I mentioned earlier, my name is Jen. I identify as cisgender female, and I prefer the pronouns “she, her, and hers.” What are your preferred pronouns?
2. How would you define your gender identity?
*Thank you. Before we move on, I wanted to provide the definition of food security so you have a better understanding of why I will be asking the questions included in this interview. “Household food security exists when all members living in your household have access to enough food for an active, healthy life at all times.”*
1. Given that definition, can you tell me about a time where you have struggled to afford enough food?
2. During times when you did not have enough food, how did you cope?
3. Tell me about a time when you feel your physical health may have suffered as a result of not being able to afford enough food.
4. Tell me about a time where you feel your mental health may have suffered as a result of not being able to afford enough food.
5. How do you think that your physical and mental health experiences influenced one another during the times when you were not able to afford enough food?
*I am now going to ask you a series of questions that aim to understand how your experiences with food security and how those experience relate to your daily life.*
1. Let us talk a little bit about your monthly expenses. Thinking back on the last month, what were your five biggest expenses?
2. Some people have told us that hormones and other health care needs are a top priority and that at times these expenses are so high that they get in the way of being able to afford food. Have you had any similar experiences?
3. When you were not able to afford enough food, how did it affect your relationships at home?
4. Please tell me about your current employment status.
*Next, I am going to ask you a series of questions that are aimed at the community in which you live and how your community might better address food security issues for gender minority people.*
1. In which city and state do you currently live?
2. How accepting is your community of gender minority people?
3. Have you ever applied for government assistance benefits such as food stamps?
4. Tell me about your experiences with local food pantries.
5. Thinking about other [transgender and gender nonconforming] people in your community, how much of an issue do you think food security is for them?
6. How do you feel your community could better support [transgender and gender nonconforming] people struggling to feed themselves or their families?

Interviews were conducted between April and June 2017, ranged from 30 to 90 min in length, and were audiorecorded. Participants' pronouns, gender identity, and state of residence were recorded. Data collection concluded when saturation was achieved.^28^

### Analysis

Following data collection from 20 interviews, qualitative transcripts were entered into QSR International's NVivo 11 for data management and analysis.^29^ Data analysis followed a hybrid method that incorporated a data-driven inductive approach^30^ and a deductive template approach using thematically grounded *a priori* codes.^31^ Four deductive codes (food security, food quality, physical health outcomes, and mental health outcomes) were developed *a priori* based on known consequences of food insecurity in the general population. The four deductive codes were applied to all sources in preliminary analysis conducted independently by two researchers (J.R. and J.P.).

Using conventional content analysis,^32^ preliminary data analysis began with reading all data repeatedly to achieve immersion. Data were then analyzed deductively using a template approach as per Crabtree and Miller.^31^ Researchers (J.R. and J.P.) also coded all transcripts inductively. The inductive coding process involved reading transcripts line by line to identify unique respondent insights within the data and then encode these insights before interpretation.^30^ The research team met weekly to discuss all coding, and specifically inductive codes, while transcripts were reviewed. Each code was discussed between researchers until any coding discrepancy was resolved.

Each member of the research team held various positions that placed us as outsiders (e.g., identifying as cisgender and never having experienced food insecurity), insiders (e.g., having experienced sexual orientation-related discrimination and having experienced food insecurity), and/or allies (e.g., community food access activists and allies to TGNC people). Outsider positions allowed us to firmly place research participants as the “experts” of their own experiences.^33^ In contrast, insider and allied positions allowed us to approach data analysis with some knowledge about the topic and general experiences faced by participants. By consistently reflecting on our personal experiences during data analysis, we were able to report unique experiences of TGNC participants as reflected by the data, rather than by our own experiences.

The final codebook described four deductive and four inductive codes, including a definition for each code and example quotes from the data, and is included as Table 2.^32^ Using Nvivo, a preliminary coding comparison query determined a high degree of agreement between coders (*Cohen's kappa* [*K*]=0.9651). Nvivo calculates percent agreement in the source data where users agree on each code's content. An average percentage agreement was calculated for 8 codes across 20 sources, where each source was given equal weight.^34^ Two overarching themes, “Experiences with Food Security” and “Health Outcomes” emerged from the coded data. A third researcher (J.J.) reviewed the data to ensure that selected text (data) appropriately represented codes, and that codes were appropriately assigned to themes.

**Table 2.** Sample Codebook

Theme	Code	Sample quote
Experiences with food security	Food security	There were days where we would go for a few days without food or even minimal—maybe a can of vegetables or something a day.
	Food quality	Luckily, I've got loads of macaroni and cheese stocked up—or ramen noodles or little things like that.
	Federal food assistance	Right now I'm getting food stamps. I just started getting them again. I was also on them about three years ago. I found it really hard to stretch them out throughout the month to make ends meet for food.
	Local food assistance	We could never actually get any food from the food pantry because the only food pantry we could get to was a Salvation Army. They're not too fond of my kind.
	Employment status	I had a job that I quickly lost, because I tried to come out as trans. They were not happy with that. I resigned.
Health outcomes	Physical health outcomes	As far as specifically in the past, eating really unhealthy food, because it was cheaper. Gained weight, and felt sick and tired all the time. Your quality of life really suffers when you put unhealthy things into your body.
	Mental health outcomes	Stress is pretty negative on your body. You start losing sleep and being more agitated easily over things that would not necessarily have agitated you before, if you have had enough food and not stressing about it.
	Support systems	I generally lean on my chosen family in times of high stress and coping with things that are hard, like not being able to afford food.

## Results

### Sample

Twenty TGNC individuals participated in the study from eight Southeastern states. Not all Southeastern states were represented in the study because no TGNC individuals residing in Alabama, Arkansas, Louisiana, or Mississippi responded to study recruitment. Demographics of the study sample are summarized in Table 3.

**Table 3.** Demographic Characteristics of Study Sample (*n*=20)

Gender identities	*n* (%)
Gender fluid	2 (10)
Gender nonconforming	1 (5)
Genderqueer	2 (10)
Nonbinary	4 (20)
Transgender female	3 (15)
Transgender male	8 (40)
States of residence	
Florida	4 (20)
Georgia	2 (10)
Kentucky	3 (15)
North Carolina	1 (5)
South Carolina	2 (10)
Tennessee	4 (20)
Virginia	3 (15)
West Virginia	1 (5)

### Theme 1: experiences with food security

This theme reports on TGNC people's experiences with food security, severity of their food security status, use of federal and local food assistance programs, and how employment status related to their level of food security. Five codes comprise this theme: food security, food quality, federal food assistance, local food assistance, and employment status.

#### Food security

Quantitatively, participants reported low-to-very low food security based on responses to the USDA prescreening module.^25^ Nineteen participants (95%) scored a 6 on the Food Security Module, indicating very low food security and 1 participant (5%) scored a 4, indicating low food security.

Participants reported not having enough food to eat and recalled frequently skipping daily meals. One participant noted, “There were days where we would go for a few days without food or even minimal—maybe a can of vegetables or something a day.” (Participant 16, Transgender Male)

To supplement limited food, participants reported seeking food from no-cost sources, including dumpsters.

“We have dived dumpsters throughout the years many times, which usually produce something good, but most of the time, dumpsters are locked up.” (Participant 20, Transgender Male)

Participants also described competing monthly financial obligations. Paying utility bills, car payments, and rent took priority, leaving participants with little income to secure adequate food.

“Rent is always paid cuz I don't wanna be homeless. The car payments are usually made on time just cuz I don't want to be embarrassed to have my car towed away and then I wouldn't have any way to get to work. I have to prioritize these things. I don't want to be homeless, definitely don't want to be jobless.” (Participant 12, Genderqueer)

The burden of paying for housing, transportation, and food also made it difficult for participants to afford health-related costs, including monthly hormones. When faced with competing costs and constrained finances, hormones were often deprioritized.

“[How] can I afford my T this month when I'm barely able to make my bills and I hardly have enough gas to get back and forth to work? Let alone I need to put milk in the fridge and get bread.” (Participant 13, Transgender Male)

Competing financial priorities also forced participants to choose between meeting daily needs, including food, and saving for gender-affirming surgeries.

“I'm gonna have to have another surgery. I was hoping that maybe I wouldn't, but it's becoming apparent to me that I need to. I hate buying food because every time I buy food I think about how it's getting—it's something I need, but it's getting in the way of something else that I need.” (Participant 8, Transgender Male)

Together, participants' narratives revealed complicated emotional and mathematical calculations where their most basic needs were pitted against each other. As meeting all of these needs was financially impossible, participants consistently engaged in a process of prioritization, which often deemed securing adequate food a lower priority.

#### Food quality

When participants were able to access food, it was often nutritionally void, processed foods. Participants reported buying the cheapest food available in an effort to have any food source at all.

“I went to the Dollar Store a lot, cuz they took my EBT card. I would just buy a lot of really cheap—like Top Ramen or canned things, pasta, just things that were cheap and that I could stretch out throughout the month as best I could.” (Participant 11, Genderqueer)

Participants were aware of the nutritional deficiencies in the food they were purchasing. However, given their limited financial capacity, many reported it came down to a choice between eating nutritionally deficient foods and not eating at all.

“I feel like a lot of times I was eating things that weren't very nutritious, because I could get ‘em for $0.99. You can get a sucker at the store for $0.25 or whatever. Those kinda things don't sustain you. They're not giving me the right protein and vitamins. I feel like for a while, I felt nauseous a lot. All the time. I was sick all the time.” (Participant 17, Gender fluid)

#### Federal food assistance

Participants reported applying for Federal Assistance Programs, such as the Supplemental Nutrition Assistance Program, but were often approved for very low monthly allowances.

“We applied for food stamps, but because we weren't married at the time and we didn't have any kids, we only got $30.00 off of that, so we'd try and make the food stretch as long as we can by buying things in bulk like rice.” (Participant 2, Nonbinary)

Others reported being denied assistance due to income earnings barely over the set allowances to qualify for federal food assistance. One participant recalled, “I'm not currently on them [food stamps]. I make 50 dollars too much to be on them right now.” (Participant 6, Nonbinary)

#### Local food assistance

Several participants noted that local food pantries were not accessible to them in their communities. One participant stated, “If you're not living in a homeless shelter, there's not much access to food pantries.” (Participant 4, Transgender Male) Among participants who noted that food pantries were available and known, almost all reported that these resources were organized by local churches or faith-based organizations. Participants reported distress when deciding to use these food pantries, as they felt uncomfortable and unwelcomed by these institutions due to their TGNC identity.

“We had a couple food banks near us, but we were iffy about going to them. Pretty much all of the things in our area are run out of conservative churches, so my wife would have to wear a binder and put her hair up. I would also have to dress more feminine than I was comfortable with at that time and just keep our heads down, hope nobody noticed anything off.” (Participant 2, Nonbinary)

In addition, participants were hesitant to use food pantries because they felt that these resources should be reserved for more vulnerable community members who may be in desperate need.

“I felt like there were people who needed a lot more than I did. I felt like I owed it to them as much as myself to look for whatever options I could find that didn't take away from people who are in greater need than I was.” (Participant 14, Transgender Female)

#### Employment status

Participants reported that food insecurity was directly related to inability to find steady employment that paid a living wage. Most participants reported being underemployed or unemployed, which substantially affected their ability to afford adequate food supplies.

“I've been constantly looking for work, but we have been on a strict rice and salt diet so it's not necessarily the best thing in the world right at the second. I have about 10 bucks every 22 days to spend on food.” (Participant 4, Transgender Male)

Several participants attributed their limited employment opportunities to employers' negative responses to their gender identity. Participants described challenges in securing employment and active discrimination in the workplace, leading to job loss—whether due to being “asked to leave” or preemptive resignation.

“I had a job that I quickly lost, because I tried to come out as trans. They were not happy with that. I resigned.” (Participant 17, Gender fluid)

In response to lacking traditional employment opportunities, participants recalled finding alternate forms of income, including engaging in sex work, to make ends meet.

“I couldn't find jobs for months on end, and that became a struggle to pay my rent and to find food, or not to find food, but to afford food. Ultimately, what I had to do was begin doing sex work in order to pay my bills, including buying food.” (Participant 6, Nonbinary)

### Theme 2: health outcomes

This theme documented how food insecurity was related to participants' physical and mental health. Three codes were identified under this theme: Physical health outcomes, mental health outcomes, and social support.

#### Physical health outcomes

Participants reported suffering substantial, negative physical health outcomes due to high food insecurity, including frequent illnesses and uncontrolled weight gain.

“[I was] eating really unhealthy food, because it was cheaper. Gained weight, and felt sick and tired all the time. Your quality of life really suffers when you put unhealthy things into your body.” (Participant 13, Transgender Male)

Some participants reported that new and existing chronic conditions, including unmanaged hypertension and cholesterol, were exacerbated by the stress of food insecurity.

“I've had kidney failure multiple times in my life. I think it may be happening again. I'm drinking nothing but hard water right now. I don't necessarily think it's doing too well. I'm physically surviving. I know that you can essentially live off of rice, but I don't feel like I'm doing the best. I've been getting colds often, which is strange. It's not like the disease thing. I think it's food related.” (Participant 4, Transgender Male)

#### Mental health outcomes

Participants also reported suffering negative mental health outcomes due to their high food insecurity, including depression, anxiety, and chronic stress.

“I was hungry 24/7. No one feels good when they're hungry. It was making me depressed. It was making me stressed. Because it was hard when you're hungry and you're trying to make it through all these things, and you're depressed, and your body isn't being given the right nutrition.” (Participant 17, Gender fluid)

Depression and stress interacted with participants' daily lives. One participant described how severe anxiety and depression arising from food insecurity negatively affected them, their intimate partner, and their responsibilities to care for a minor child.

“We've both been really stressed out, really anxious, really depressed about it. There have been moments where we just sat and cried about it. Her son doesn't, he doesn't understand that he can't eat everything on the house all the time because we don't have the money to buy food all the time.” (Participant 12, Genderqueer)

Despite severe stress and poor mental health arising from food insecurity, participants reported a great deal of resiliency. Several participants described strategies used to buffer stress, including practicing mindfulness and breathing techniques. Participants described being optimistic that their situation would improve, even in times of great adversity.

“I guess we're both pretty resilient. I grew up living kind of like that. It's kind of revisiting a dark moment. We just try to continue to be positive and hope that she'll get a job and now she has one luckily…We just try to remain positive and not let that affect her son and things like that, but we don't always eat.” (Participant 12, Genderqueer)

#### Support systems

Participants reported high food insecurity caused them emotional distress that strained relationships with other members of their household. Stress that participants experienced with their intimate partners was of particular concern, as it attacked and weakened their mental health and primary social support system.

“I find myself getting upset and angry because I'm the primary money-maker in the house, if that's what you call it. [My partner] and I have gotten into arguments over food like, ‘Oh, you're eating too much,’ or ‘You didn't let the milk last long enough’ or things like that.” (Participant 12, Genderqueer)

Conversely, several participants noted that the shared experience of food insecurity led them to grow closer to their partners and rely on each other during difficult times.

I feel like it's brought us closer together. I feel like kind of working through these issues together and being there for each other kind of immediately at low points in our life has created this bond and definitely kind of shown each other our true colors right from the get go. I think, in a way, it's made our relationship really strong… (Participant 17, Gender fluid)

Participants reported that relying on nuclear family members for financial support or food assistance was not always an option. Multiple participants described being rejected by and ostracized from family after coming out about their sexual orientation and/or gender identity.

“Her single mom kicked her out on her 18th birthday for being—I guess you could say—gay. Then, when I came out as trans, I haven't spoken to my family since then. We weren't able to get any sort of family support.” (Participant 20, Transgender Male)

Alternatively, participants leaned on “chosen family” or close friends. These individuals provided both emotional support and tangible resources, including food and connections to food assistance programs.

“I generally lean on my chosen family in times of high stress and coping with things that are hard, like not being able to afford food. That usually helps in terms of just alleviating the stress and also finding resources to get on things like food stamps or various things.” (Participant 6, Nonbinary)

## Discussion

Across existing studies, gender and socioeconomic inequality have been documented as intersectional social determinants of food insecurity.^35,36^ TGNC participants in our study repeatedly described experiencing gender-based stigma that impacted their financial stability and, as a result, severely limited their ability to afford adequate food. It is well documented that TGNC people experience multilevel discrimination due to their gender identity.^9^ Approximately 63% of TGNC people report serious acts of discrimination—including joblessness, homelessness, denial of services, and violence—due to gender bias.^9^

Participants in our study reported substantial challenges in finding steady employment that paid a living wage due to transphobia and gender bias. TGNC participants described multiple interpersonal and institutional minority stressors, including being turned down for job interviews, denied opportunities for promotion, and losing employment after coming out as transgender in the workplace. To make up for lost income and in the face of food insecurity, TGNC participants turned to “underground” sources of income, including sex work. Among vulnerable, food-insecure populations, engaging in sex for money is a documented theme.^37–39^ Reliance on sex work to increase food security is especially concerning, as the illegality and stigma associated with sex work further marginalizes food-insecure and TGNC people and increases their risk for harm.^39^

For food-insecure people in the general population, local food pantries are widely utilized sources of food^19^; however, the vast majority of U.S. food pantries are organized and operated by faith-based organizations.^40^ Religiously affiliated food pantries presented a substantial barrier for TGNC people in our study. Participants feared experiencing community-level minority stress in the form of gender-based discrimination from “conservative” or “anti-LGBT” religious groups who organize food pantries. In turn, fear kept participants from seeking much-needed food assistance within their local communities. This finding contrasts qualitative findings among food-insecure adults in which faith-based communities are reported as a source of regular food assistance.^35,36^ As reported by others,^41^ we also found that TGNC participants who accessed food pantries described going “stealth” and dressing in contrast to their gender identity to avoid transphobia. While going stealth facilitated access to local food sources for participants in our study, it resulted in physical discomfort and psychological distress.

Participants also reported alternative food-seeking practices. These included searching for food in dumpsters, frequently skipping meals, or severely limiting dietary options. Similar strategies have been reported in the broader literature among low-income and food-insecure adults.^36,42,43^ In addition, nuclear family food assistance was not always a viable option. As such, respondents reported relying on “chosen family,” or close friends, as sources for food assistance.

TGNC people in our study described feeling undeserving of local food assistance. Specifically, they were concerned that local food pantries were designed for more vulnerable community members. Participants reported purposefully not seeking local food assistance to avoid taking away critical resources from those in dire need. From our understanding, this is a unique finding.^36^ In other studies, participants experienced concern about taking food away from vulnerable groups when choosing to use food pantries; however, these concerns did not prevent use of these resources.

One explanation for our finding is that in the context of a cissexist, binary gender identity system where “normal” is defined as either “man” or “woman,” TGNC people are seen as “abnormal” or “less than” because of their transgender or gender nonconforming identity.^44^ This gender-based stigma may exacerbate or generate feelings of “unworthiness” among TGNC people who are also impoverished and food insecure, which may explain why participants in this study felt undeserving of food assistance. It may also be that feeling unworthy is an emotion faced by food-insecure people in general.

Participants reported relying on nutritionally deficient food sources. This is concerning as these foods can contribute to poor physical health and chronic disease. Of particular concern were participants' reports of hypertension and high cholesterol, which they attributed to stress and inadequate nutrition arising from food insecurity. Recent population-based studies indicate that gender nonconforming, transgender females,^45^ and transgender males^46^ exhibited disparities in cardiovascular disease. While larger studies are needed, it may be that food insecurity is an important driver of cardiovascular disease risks.

Psychosocial consequences of food insecurity are not unique to TGNC people. Experiencing hunger, negotiating food assistance programs, and acquiring food are universal experiences cited as sources of stress among food-insecure people in the general literature and in this study.^35,42,47^ However, for TGNC people, each source of food-related stress intersected with unique minority stressors arising from cissexism and transphobia. Multilevel gender-based discrimination and victimization by family members, at work, and in the local food assistance community, were layered upon TGNC peoples' experience of food insecurity.

Yet, even facing compounded minority stress and extreme food insecurity, TGNC participants exhibited extraordinary resilience. Meyer^48^ describes resilience as the ability to successfully adapt and cope with acute and chronic minority stressors. It comprises multiple biopsychosocial processes that buffer the effects of stress and promote health.^49^ While their strategies to cope with stress varied, resilience was the thread that ran through each person's experience. As summed by one participant, “You have to gut yourself. It's an emotional thing that you have to dig through and find everything that you are and just come out and be happy.” Despite cumulative minority stress, poverty, and food insecurity, TGNC participants were resilient and coped.

## Limitations

This study attempted to capture the experiences of food insecure TGNC people in the Southeast U.S; however, not all Southeastern U.S. states were represented. One explanation is that TGNC people residing in states that were not represented are experiencing some of the worst contexts for food insecurity and gender-based discrimination.^18,19^ As such, they may have been less likely to participate, given contextual stressors. In addition, self-reported gender identity and level of food security were the only demographic characteristics collected in this study. Thus, it is impossible to determine whether TGNC people of diverse backgrounds are represented. Finally, the sample size (*n*=20) for this study was small, but saturation was achieved.

## Implications and Future Directions

Several public health solutions could be implemented in the Southeast U.S. to alleviate issues of food insecurity among TGNC people. Structurally, Federal- or state-level legislation must be established to protect TGNC people from employment discrimination. In much of the Southeast United States, state laws do not guard against employment discrimination for TGNC individuals.^18^ Employment discrimination decreases opportunities for permanent, stable employment, thereby systematically and unjustly increasing risk for food insecurity among TGNC people.

Community-based solutions that increase access to safe and affirming resources for food insecure TGNC people are also warranted. Local food pantries could partner with LGBT community organizations to create TGNC-affirming food pantries and/or assist existing food pantries in becoming TGNC affirming. Through such partnerships, food pantry staff and leadership could be educated about food insecurity among TGNC people and trained in strategies to make food pantries safer for and more accessible to this population.

## Abbreviations Used

LGBTQ: Lesbian, Gay, Bisexual, Transgender, Questioning
PI: Principal Investigator
TGNC: Transgender and gender nonconforming
USDA: United States Department of Agriculture
USTS: U.S. Transgender Survey
